# Protective Effects of *Scolopendra* Water Extract on Trimethyltin-Induced Hippocampal Neurodegeneration and Seizures in Mice

**DOI:** 10.3390/brainsci9120369

**Published:** 2019-12-12

**Authors:** Yun-Soo Seo, Mary Jasmin Ang, Byeong Cheol Moon, Hyo Seon Kim, Goya Choi, Hye-Sun Lim, Sohi Kang, Mijin Jeon, Sung-Ho Kim, Changjong Moon, Joong Sun Kim

**Affiliations:** 1 Herbal Medicine Resources Research Center, Korea Institute of Oriental Medicine, 111, Geonjae-ro, Naju-si 58245, Jeollanam-do, Korea; sys0109@kiom.re.kr (Y.-S.S.); bcmoon@kiom.re.kr (B.C.M.); hs0320@kiom.re.kr (H.S.K.); serparas@kiom.re.kr (G.C.); qp1015@kiom.re.kr (H.-S.L.); 2 College of Veterinary Medicine and BK21 Plus Project Team, Chonnam National University, Gwangju 61186, Korea; ang.maryjasmin@gmail.com (M.J.A.); shrloveu@gmail.com (S.K.); mjori@hanmail.net (M.J.); shokim@chonnam.ac.kr (S.-H.K.)

**Keywords:** *Scolopendra subspinipes*, trimethyltin, neuronal degeneration, hippocampus, seizure

## Abstract

Trimethyltin (TMT) is an organotin compound with potent neurotoxic action characterized by neuronal degeneration in the hippocampus. This study evaluated the protective effects of a *Scolopendra* water extract (SWE) against TMT intoxication in hippocampal neurons, using both in vitro and in vivo model systems. Specifically, we examined the actions of SWE on TMT- (5 mM) induced cytotoxicity in primary cultures of mouse hippocampal neurons (7 days in vitro) and the effects of SWE on hippocampal degeneration in adult TMT- (2.6 mg/kg, intraperitoneal) treated C57BL/6 mice. We found that SWE pretreatment (0–100 μg/mL) significantly reduced TMT-induced cytotoxicity in cultured hippocampal neurons in a dose-dependent manner, as determined by lactate dehydrogenase and 3-[4,5-dimethylthiazol-2-yl]-2,5-diphenyltetrazolium bromide assays. Additionally, this study showed that perioral administration of SWE (5 mg/kg), from −6 to 0 days before TMT injection, significantly attenuated hippocampal cell degeneration and seizures in adult mice. Furthermore, quantitative analysis of Iba-1 (Allograft inflammatory factor 1)- and GFAP (Glial fibrillary acidic protein)-immunostained cells revealed a significant reduction in the levels of Iba-1- and GFAP-positive cell bodies in the dentate gyrus (DG) of mice treated with SWE prior to TMT injection. These data indicated that SWE pretreatment significantly protected the hippocampus against the massive activation of microglia and astrocytes elicited by TMT. In addition, our data showed that the SWE-induced reduction of immune cell activation was linked to a significant reduction in cell death and a significant improvement in TMT-induced seizure behavior. Thus, we conclude that SWE ameliorated the detrimental effects of TMT toxicity on hippocampal neurons, both in vivo and in vitro. Altogether, our findings hint at a promising pharmacotherapeutic use of SWE in hippocampal degeneration and dysfunction.

## 1. Introduction

Centipedes, especially *Scolopendra subspinipes* (Leach, William, 1815) and *Scolopendra subspinipes mutilans* (syntype NHMW (Naturhistorisches Museum Wien) of *S. mulitans* Koch, 1878), have been used in oriental medicine to treat neuroinflammatory conditions including spastic diseases such as spasms, childhood convulsions, and seizures [1,2,3]. In Korea, China, and other Asian regions, *Scolopendra* water extracts (SWE) have been used to treat neurodegenerative diseases [4], neuropathic pain [5], and inflammatory diseases [6,7]. Although SWE has been used for the treatment of patients with nerve injuries accompanied by neuroinflammation, the molecular mechanisms of the therapeutic actions of SWE are not yet clear. *Scolopendra* pharmacopuncture is typically used to treat entrapment neuropathies and convulsive diseases [6]; however, no study to date has examined the therapeutic effects of SWE using a seizure model.

Trimethyltin (TMT) is an organotin compound that exhibits potent neurotoxic actions, characterized by selective neuronal destruction in the limbic system [8,9]. Historically, TMT has been widely used, for instance, in the plastics industry as a stabilizer in the manufacture of plastic that requires heating and in the agricultural field as a fungicide. Several cases of human intoxication by TMT in workplaces have been reported [10]. Humans accidentally exposed to TMT develop a syndrome characterized by seizures, disorientation, and confusion [8,10]. In experimental animals, TMT administration induces severe hippocampal damage and consequent behavioral alterations such as ataxia, aggression, tail mutilation, vocalization, and seizures [11,12]. These behavioral features mimic those of individuals occupationally or accidentally exposed to TMT, and the similar temporal correlation between the time of cell death and the onset of the seizure behavior may suggest a causal relationship between TMT-induced hippocampal damage and seizure behavior [13,14]. Thus, the pattern of hippocampal damage in rats and the subsequent seizure behavior caused by TMT exposure make this chemical an ideal research tool to study neurodegeneration [13]. In this study, we explored the protective effects of SWE using in vitro and in vivo mouse models of TMT-induced hippocampal degeneration and seizures.

## 2. Materials and Methods

### 2.1. Preparation of SWE

Dried S. *subspinipes* specimens were purchased from HANDONG HERB Co. (Seoul, Korea). The medicinal materials were authenticated morphologically by Dr. Goya Choi at the Korea Institute of Oriental Medicine (KIOM), Daejeon, Korea. Voucher specimens were deposited in the Korean Herbarium of Standard Herbal Resources (herbarium code KIOM, specimen no. 2–18–0113) at KIOM. The extract from *S. subspinipes mutilans* (750 g) was prepared by a 3 h reflux extraction using 15 L of water as solvent (100 ± 2 °C). The solution was then concentrated under reduced pressure using a vacuum evaporator. The concentrated extract was lyophilized using a freeze dryer, which produced 155.01 g of lyophilized powder (yield 20.67%).

### 2.2. Primary Hippocampal Cell Culture and SWE Treatment

The primary culture of hippocampal neurons has been previously described [12,15]. Briefly, hippocampi were dissected from C57BL/6 mice pups at 17–18 gestational days. The neurons were then dissociated and subsequently cultured in vitro, following standard procedure. All cultures were maintained at 37 °C and 5% CO_2_. The primary hippocampal cultured neurons were treated with 50 mM of TMT after 7 days in vitro (DIV) and assayed 24 h after treatment. To evaluate the cytoprotective effects of SWE on TMT-induced damage of mature hippocampal neurons, SWE (0–100 μg/mL) was added 1 h before TMT treatment (*n* = 3 cultures per condition).

### 2.3. Cytotoxicity and Cell Viability Examination

Cytotoxicity was tested using a lactate dehydrogenase (LDH) release assay. An LDH cytotoxicity assay kit from BioVision (Mountain View, CA, USA) was used in the way recommended by the manufacturer. The optical density values were quantified by measuring absorbance at a wavelength of 450 nm using a microplate reader (EMax, Molecular Devices, Sunnyvale, CA, USA) [12]. 

Cell viability was evaluated using the 3-[4,5-dimethylthiazol-2-yl]-2,5-diphenyltetrazolium bromide (MTT; Sigma–Aldrich, St. Louis, MO, USA) assay. This assay is based on the reduction of MTT by living cells to yield a soluble formazan product that can be colorimetrically detected. Briefly, MTT was added at 0.5 mg/mL in the culture medium of cells growing in 4-well plates. After a 2 h of incubation at 37 °C with 5% CO_2_, the resulting formazan crystals were dissolved in MTT solubilization solution (Sigma–Aldrich) and quantified using an Emax spectrophotometer (Molecular Devices) by measuring the absorbance at 595 nm. The background absorbance at 690 nm was subtracted [12].

### 2.4. Preparation of SWE

Specific pathogen-free male C57BL/6 mice (of age 8 weeks and weight 20–25 g) were purchased from Orient Bio, Inc. (Seoul, Korea) and used after a 2-week period of quarantine and acclimatization. All experiments and protocols used in this study were approved by the Institutional Animal Care and Use Committee of Chonnam National University (CNU IACUC-YB-2018-69), and the animals were cared for in accordance with the National Institute of Health Guide for the Care and Use of Laboratory Animals.

TMT (Wako, Osaka, Japan) was dissolved in sterilized 0.9% saline. To assess the effects of TMT-induced seizures in mouse hippocampi, mice were sacrificed, and the brains were dissected 3 days after a single intraperitoneal (ip) injection of vehicle (0.9% saline) or 2.6 mg/kg of TMT solution (*n* = 6 mice per group), as adapted from a previously described protocol [13]. All the animals were euthanized by decapitation after 4 days after TMT treatment.

### 2.5. Drugs, Treatments, and Tissue Sampling

To assess the effects of SWE on TMT-induced injury, mice were administered SWE (50 mg/kg) or vehicle (0.9% saline) periorally (po) for one week, before being given 2.6 mg/kg TMT (*n* = 6 mice per group). Their behaviors were observed for 3 consecutive days after TMT treatment. 

To assess the effects of SWE treatment on histological changes in the hippocampus, mice were euthanized 4 days after treatment. The samples were embedded in paraffin wax after fixation with 10% formalin solution, using routine protocols (*n* = 3 mice per group).

### 2.6. Seizure Scoring

Seizure behavior tests were performed in a bright box (40 × 40 cm, lit at 250 lux). Behavioral changes gave scores ranging between score 1 (aggression), score 2 (weak tremor), score 3 (systemic tremor), score 4 (tremor and spasmodic gait), and score 5 (death) [12,16].

### 2.7. Immunohistochemistry: Microglia and Astrocyte Staining

Coronal sections (4 µm thick) were cut and deparaffinized using routine protocols before being incubated with a monoclonal anti-Iba-1 antibody (1:500 dilution; Thermo Fisher Scientific, Waltham, Massachusetts (MA), USA) or a rabbit anti-GFAP antibody (1:1000 dilution; Dakocytomation, Glostrup, Denmark). Primary antibody binding was detected with biotinylated horse anti-mouse or goat anti-rabbit IgG (Vector Elite kit; Burlingame, CA, USA), respectively. Immunoreactivity was measured using an avidin–biotin peroxidase complex (Vector Elite kit). The peroxidase reaction was developed using the diaminobenzidine substrate assay (Vector Elite kit). The slides were counterstained with Harris’ hematoxylin, before being mounted with Canada balsam (Sigma-Aldrich) [17].

### 2.8. Fluoro-Jade C (FJC) Staining

To detect neuronal death, histofluorescent staining was performed, according to a previously described method [17,18]. In brief, sections were first transferred to a solution of 0.06% potassium permanganate and then to a 0.0001% FJC (Millipore) staining solution. FJC is a highly anionic and acidic marker with an affinity for degenerating neurons. After FJC staining, sections were counterstained with 4′,6-diamidino-2-phenylindole, dihydrochloride (DAPI) to label nuclei, before being mounted onto microscope slides. FJC-stained sections were examined by immunofluorescence microscopy, using a BX-40 apparatus (Olympus, Tokyo, Japan) with an eXcope X3 (Olympus) digital camera. 

### 2.9. Statistical Analyses

The data are reported as the mean ± SE and were analyzed using one-way analysis of variance (ANOVA) followed by a Student-Newman-Keuls post-hoc test for multiple comparisons. In all cases, a *p* value < 0.05 was considered significant.

## 3. Results

### 3.1. SWE Protects Hippocampal Cultured Neurons against TMT-Induced Neural Injury

**Figure 1:**
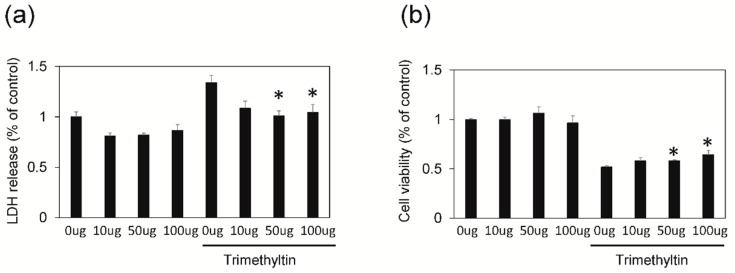
Protective effects of a *Scolopendra* water extract (SWE) on trimethyltin (TMT)-induced cytotoxicity. SWE treatment reduced the cytotoxic effects of TMT on hippocampal neurons. lactate dehydrogenase (LDH) assay (**a**) and 3-[4,5-dimethylthiazol-2-yl]-2,5-diphenyltetrazolium bromide (MTT) assay (**b**). Values are reported as mean ± SE, * *p* < 0.05.

On the basis of our previous study [19], we assessed whether SWE prevented or ameliorated TMT-induced injury in hippocampal cells at 12 DIV by LDH release tests. We found that, while treatment with TMT (5 mM) alone increased LDH release from cultured hippocampal neurons compared to saline-treated controls, at 24 h after treatment (*n* = 3 cultures per condition; *p* < 0.05; see Figure 1a), pretreatment of cultured neurons with SWE (0–100 mg/mL) significantly inhibited TMT-induced cytotoxicity in a dose-dependent manner (*n* = 3 cultures per condition; Figure 1a). This result suggested a protective action of SWE on TMT-induced neuronal death. To further confirm our data, an MTT assay was performed in the primary hippocampal cultures 24 h after treatment. As shown in Figure 1b, SWE treatment remarkably reduced TMT-induced neuronal cell death (*n* = 3 cultures per condition). Thus, data from two different, but complementary, assays measuring cytotoxicity and cell viability consistently revealed that SWE pretreatment significantly decreased TMT-induced neuronal cell death in cultured, mature hippocampal cells.

### 3.2. SWE Protects from TMT-Induced Seizures

**Figure 2:**
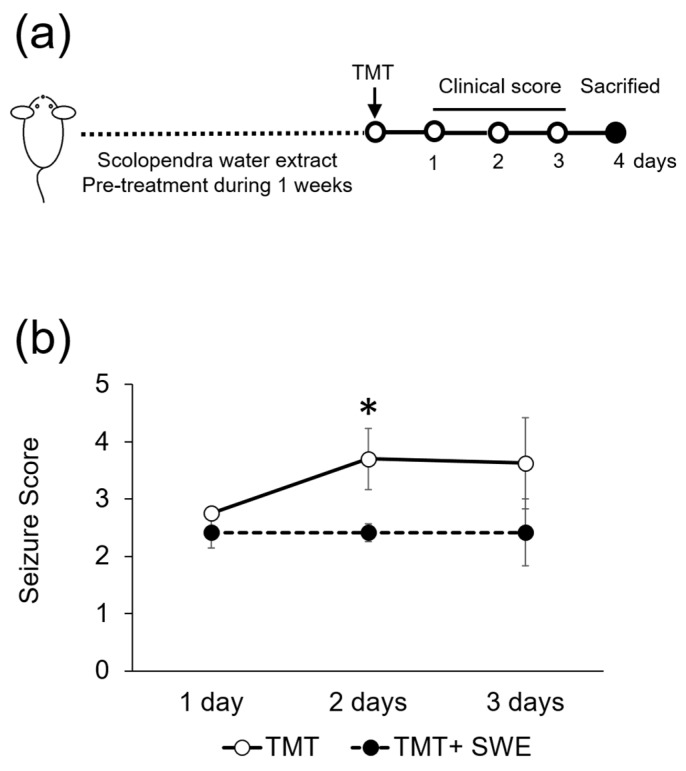
Protective effects of SWE on seizure symptoms in TMT-treated mice. (**a**) Schematic diagram of drug treatment, tissue preparation, and behavioral test. (**b**) SWE treatment ameliorated TMT-induced seizure behaviors (*n* = 6 mice per group). Values are reported as mean ± SE, * *p* < 0.05.

After TMT intoxication, symptoms such as tremors, seizures, and aggressive behavior increased rapidly, for a period of 2 to 3 days in mice (Figure 2). However, the TMT-induced seizure behavior score in SWE-pretreated mice was significantly lower than in non-pretreated controls (*n* = 6 mice per group; Figure 2). The seizure behaviors in both TMT- and SWE + TMT-treated mice had disappeared on day 6 after TMT treatment.

### 3.3. SWE Treatment Results in Decreased Levels of Iba-1- and GFAP-Positive Cells in the Hippocampi of TMT-Treated Mice

**Figure 3:**
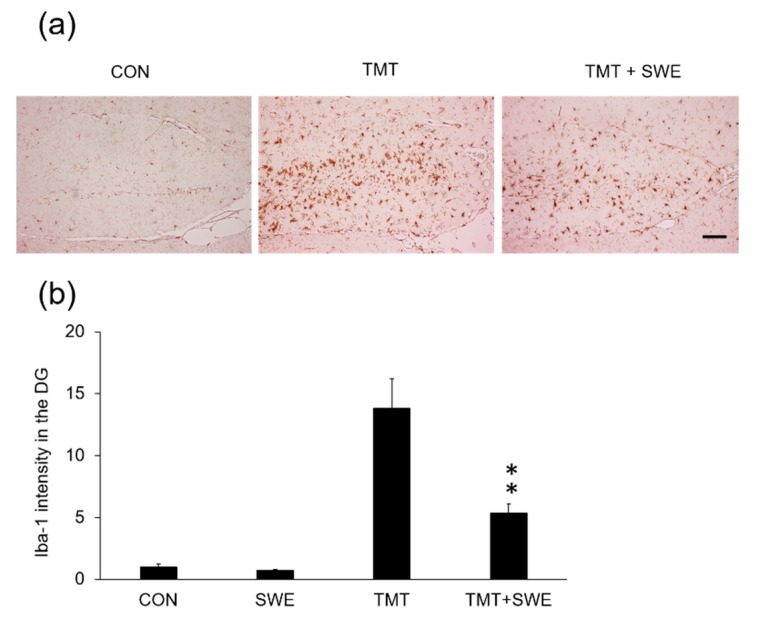
Protective effects of SWE on microglial cell activation in the hippocampus 4 days after TMT treatment. Representative images (X 200) showing microglial Iba-1 immunostaining in untreated control, TMT control, and SWE + TMT group (**a**). The graph (**b**) depicts the relative intensity of Iba-1 positive cells in the dentate gyrus (DG) of the hippocampus sections. Values are reported as mean ± SE, *n* = 3 in each group. * *p* < 0.05. Scale bar = 250um.

**Figure 4:**
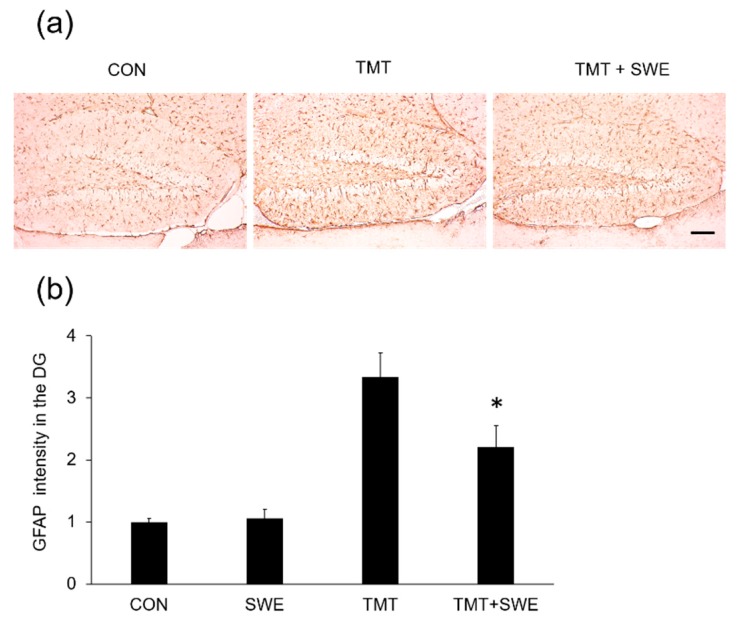
Protective effects of SWE on astrocyte levels in the DG, 4 days after TMT treatment. Representative images (X 200) showing GFAP immunostaining in untreated control, TMT control, and SWE + TMT group (**a**) The graph (**b**) depicts the relative number of GFAP-positive cells per dentate gyrus in the hippocampus sections. Values are reported as mean ± SE, *n* = 3 in each group. * *p* < 0.05. Scale bar = 250um.

To investigate the effects of SWE on microglia and astrocyte activation during TMT-elicited neuroinflammation, we used immunohistochemical assays. Specifically, we assessed the density of Iba-1- and GFAP-positive cells in the hippocampus, 4 days after TMT treatment (*n* = 3 mice per group). We found that Iba-1 positive cells (Figure 3a) were constitutively expressed in the dentate gyrus (DGs) of adult hippocampi in control mice and that the number of Iba-1-positive cells significantly increased in the hippocampus 3 days after TMT treatment (Figure 3b). However, pretreatment with SWE resulted in a significantly reduced number of Iba-1-positive cells, when compared with the TMT-treated group (*p* < 0.05). Similarly, GFAP-positive cells significantly increased in the hippocampus 4 days after TMT treatment, and SWE pretreatment markedly reduced the number of GFAP-positive cells (*p* < 0.05 vs. TMT-treated mice) (Figure 4a,b).

### 3.4. SWE Treatment Ameliorates Hippocampal Neuron Degeneration in TMT-Treated Mice

**Figure 5:**
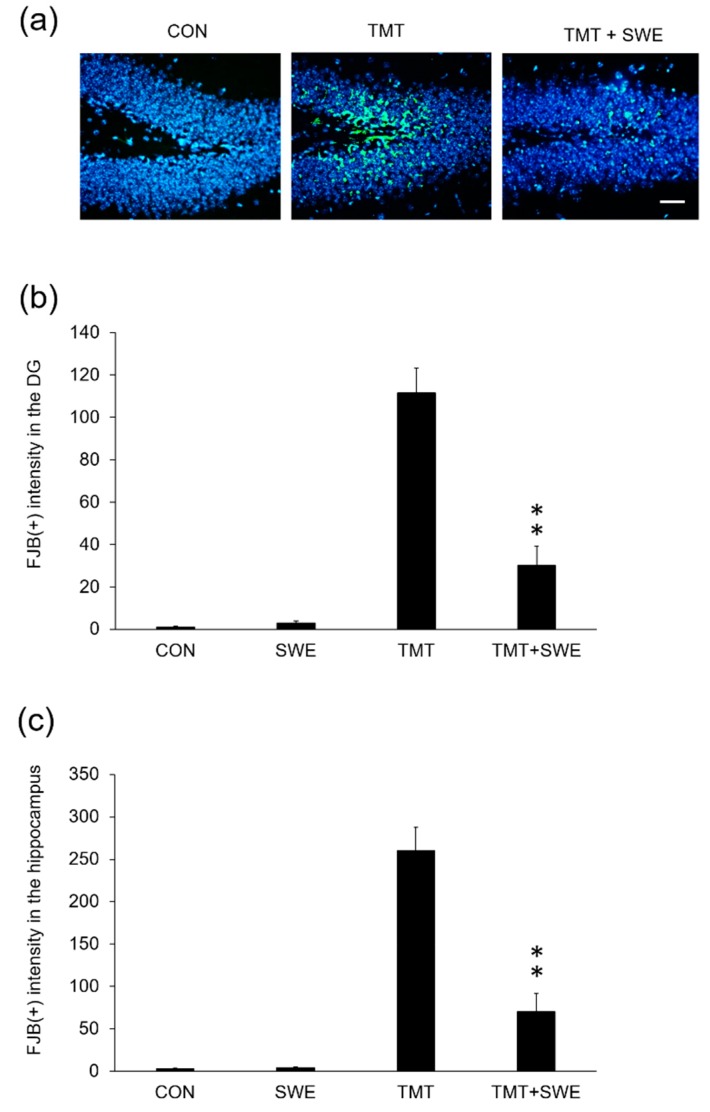
Protective effects of SWE on the incidence of Fluoro-Jade (FJC)-1-positive dead cells in the DG of the hippocampus, 4 days after TMT treatment. Representative images (X 200) showing degenerating cells stained with FJC stain in untreated control, TMT control, and SWE + TMT group (**a**). All tissues were collected at 4 days after TMT treatment. The graph (**b**) depicts the intensity of FJC-1-positive cells per dentate gyrus (**b**) and entire hippocampus (**c**) in the brain sections. Values are reported as the mean ± SE, n = 3 in each group. * *p* < 0.05. Scale bar = 500um.

To evaluate whether there was a correlation between histological data and SWE protective action on neurons in vivo, we examined degenerating neurons in the hippocampus of TMT-treated mice (*n* = 6 mice per group) using FJC staining [20]. FJC-positive neurons were measured 4 days after TMT treatment. We found that the number of FJC-positive cells in TMT-treated mice was significantly increased compared to that in vehicle-treated mice and that pretreatment with SWE markedly reduced the number of degenerating neurons compared to TMT-treated mice (*p* < 0.05) (Figure 5a,b).

## 4. Discussion

Although the pathological mechanisms that underlie seizures remain unclear, there is evidence that brain inflammation is implicated. Epilepsy has been linked to increased levels of inflammatory mediators in the brain, and brain inflammation might contribute to the onset and perpetuation of seizures in a variety of epilepsies. However, new experimental and clinical research is required to discover and validate novel therapeutic anti-inflammatory approaches to ameliorate seizures and modify their underlying pathophysiology [21]. In the present study, we found that SWE pretreatment attenuated TMT-induced hippocampal damage. Specifically, SWE treatment significantly protected hippocampal neurons from TMT cytotoxicity, as demonstrated in our in vitro experiments using primary hippocampal cell cultures. In addition, SWE treatment reduced seizure behavior and the number of microglial cells, astrocytes, and degenerating neurons in the hippocampus of TMT-treated mice. 

TMT is a neurotoxic reagent that induces neurodegeneration of the mammalian limbic system. Neurodegeneration caused by TMT toxicity results from complex events including neuroinflammation, glutamate excitotoxicity, intracellular calcium overload, oxidative stress, mitochondrial dysfunction, and impaired neurotransmission [22]. Mice exposed to TMT suffer spontaneous seizure behaviors after TMT injection. The course of clinical symptoms in mice is consistent with the histopathological changes in the mouse hippocampus [23]. The present study demonstrated that pretreatment with SWE prevented seizure behavior induced by TMT by protecting hippocampal cells from degeneration. An inflammatory response contributes to disease pathogenesis in various injury models of the nervous system, including TMT-induced neurotoxicity [24]. Activated microglia and astrocytes participate in inflammation in the nervous system by stimulating phagocyte proliferation and releasing inflammatory cytokines, which are directly toxic to neurons [24,25]. Furthermore, after brain injury, activated astrocytes become swollen, with enlarged cell bodies and projections into the damaged area and increased GFAP expression [26]. Although microglia are a major source of inflammatory cytokine production, astrocytes also secrete cytokines in response to brain damage. Microglia and astrocytes are differentially activated in temporal and spatial patterns and can regulate each other via cellular crosstalk [27].

Neurologic inflammation may result in seizures, suggesting that inflammation may be involved in epileptogenesis. After TMT treatment, activated glial cells release various inflammatory substances, including inducible NO synthase, cyclooxygenase-2, and chemokines, as well as diverse cytokines such as IL (Interleukin)-6, IL-17 and IL-17 receptor (IL-17R) axis, tumor necrosis factor α (TNF-α), and transforming growth factor β (TGF-β), responding to TMT-induced neural injury [28,29]. Clinical findings support the role brain inflammation has in the pathogenesis of seizures. In addition, emerging proof-of-concept studies have shown the clinical efficacy of target-specific anti-inflammatory interventions in epilepsy with differing etiologies. As discussed below, there is a need for biomarkers and novel clinical trial designs for anti-inflammatory therapies that act very differently from standard antiepileptic drugs [30]. A previous study showed that lithium attenuated TMT-induced hippocampus damage with seizures and memory loss in mice model. This suggested an anti-inflammatory role of autophagy block and GSK-3/β-catenin signaling [17,31]. *Scolopendra* treatment has been reported to have many biochemical and physiological effects [32]. SWE contains various amino acids, such as aspartate, arginine, alanine, leucine, and lysine [33], while the composition of centipede venom includes serotonin, histamine, lipids, polysaccharides, and polypeptides [34]. A previous study demonstrated that SWE decreased the permeability of abdominal blood capillaries and ear inflammation. It also decreased the pain threshold in mice during hot-plate and writhing tests [35]. In addition, a previous study reported anti-inflammatory effects of *Scolopendra* in an Alzheimer’s disease model [36]. Similarly, another study showed that SWE administration attenuated the loss of motor neurons in the spinal cord of an amyotrophic lateral sclerosis model [37].

To evaluate if the protective effects of SWE on the hippocampus were related to a decreased activation of microglia and astrocytes, we assessed histological changes after administration of SWE or control in TMT-treated mice. Previous work using immunohistochemical analysis revealed activated morphologies of microglia and astrocytes after TMT treatment [16,24]. Moreover, Iba-1-positive microglial cells were greatly upregulated in the granular cell layer of the hippocampal DG 4 days after treatment, and GFAP-positive astrocytes also reached a maximum number 4 days after TMT treatment, presenting hypertrophied forms [16]. Notably, our results showed that the administration of SWE prevented TMT-induced activation and expansion of microglia and astrocytes. Furthermore, SWE pretreatment prevented the neurodegeneration of the hippocampus induced by TMT. Thus, our work indicates that SWE attenuates hippocampal injury by blocking the activation of microglia and astrocytes.

We additionally performed a post-treatment study of SWE. Post-treatment with SWE did not appear to have any significant therapeutic effects (Supplementary Figure S1). Pretreatment can be considered effective when used in complementary medicine. In a further study, we will be able to verify these mechanisms as well as if a therapeutic effect is exerted by SWE post-treatment. Although pretreatment is of small clinical meaning, it can be considered effective in the case of complementary medicine. 

## 5. Conclusions

Altogether, we conclude that SWE treatment had an anti-neuroinflammatory effect in vivo by preventing the activation of microglia and astrocytes elicited by TMT in the DG. Accordingly, we also found that the SWE-induced reduction of immune cell activation was coupled with a significant reduction in hippocampal cell death and a consequent significant improvement in TMT-induced seizure behavior. Although the mechanisms regarding the protective effects of SWE remain unclear, this study provides evidence that SWE might have a promising therapeutic effect in the treatment of neuronal diseases such as epilepsy.

## Supplementary Materials

The following are available online at https://www.mdpi.com/2076-3425/9/12/369/s1, Figure S1: Protective effects of SWE on seizure symptoms, in TMT-treated mice.
