# Supplementary material for: Protective Effects of Scolopendra Water Extract on Trimethyltin-Induced Hippocampal Neurodegeneration and Seizures in Mice
Source: Brain Sci. 2019 Dec 12;9(12):369. doi: 10.3390/brainsci9120369 (PMC6955677; doi:10.3390/brainsci9120369)
Supplement: Supplementary file 1 [file brainsci-09-00369-s001.pdf]

Supplementary materials

Post treatment protocol

To assess the effects of SWE on TMT-induced injury, mice were administered SWE (50 mg/kg) periorally (p.o) for 4day daily, after being given 2.6 mg/kg TMT ($n = 6$ mice per group). Behaviors were observed for 3 consecutive days after TMT treatment (Supplementary Figure S1).

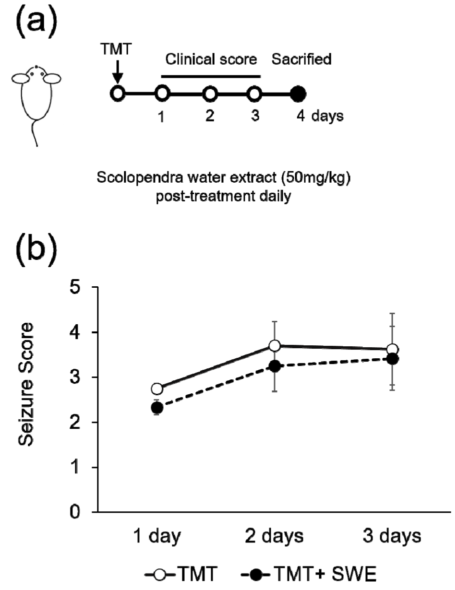

Supplementary Figure S1. Protective effects of SWE on seizure symptoms, in TMT-treated mice. (**a**) Schematic diagram of drug treatment, and behavioral test. (**b**) SWE treatment did not have effect on the TMT-induced seizure behaviors ($n = 6$ mice per group). Values are reported as mean ± SE * $p < 0.05$.
